# Single-Point Mutations within the Coxsackie B Virus Receptor-Binding Site Promote Resistance against Soluble Virus Receptor Traps

**DOI:** 10.1128/JVI.00952-20

**Published:** 2020-09-15

**Authors:** Sandra Pinkert, Anja Kopp, Vanessa Brückner, Henry Fechner, Antje Beling

**Affiliations:** a Charité–Universitätsmedizin Berlin, corporate member of Freie Universität Berlin, Humboldt-Universität zu Berlin, and Berlin Institute of Health (BIH), Institute of Biochemistry, Berlin, Germany; b Institute of Biotechnology, Department of Applied Biochemistry, Technische Universität Berlin, Berlin, Germany; c DZHK (German Centre for Cardiovascular Research), Berlin, Germany; University of Texas Southwestern Medical Center

**Keywords:** antiviral agents, coxsackievirus, drug resistance evolution, picornaviruses, soluble receptors

## Abstract

Coxsackie B viruses (CVB) cause a wide spectrum of diseases, ranging from mild respiratory syndromes and hand, foot, and mouth disease to life-threatening conditions, such as pancreatitis, myocarditis, and encephalitis. Previously, we and others found that the soluble virus receptor trap sCAR-Fc strongly attenuates CVB3 infection in mice. In this study, we investigated whether treatment with sCAR-Fc results in development of resistance by CVB3. Two CVB3 strains (CVB3-H3 and CVB3 Nancy) were passaged in HeLa cells in the presence of sCAR-Fc. The CVB3-H3 strain did not develop resistance, whereas two populations of CVB3 Nancy mutants emerged, one with complete (CVB3_M_) and one with partial (CVB3_K_) resistance. DNA sequence alignment of the resistant virus variant CVB3_M_ with CVB3 Nancy revealed an amino acid exchange from Asn(N) to Ser(S) at position 139 of the CVB3 capsid protein VP2 (N2139S), an amino acid predicted to be involved in the virus’s interaction with its cognate receptor CAR. Insertion of the N2139S mutation into CVB3-H3 by site-directed mutagenesis promoted resistance of the engineered CVB3-H3_N2139S_ to sCAR-Fc. Interestingly, development of resistance by CVB3-H3_N2139S_ and the exemplarily investigated CVB3_M_-clone 2 (CVB3_M2_) against soluble CAR did not compromise the use of cellular CAR for viral infection. Infection of HeLa cells showed that sCAR-Fc resistance, however, negatively affected both virus stability and viral replication compared to that of the parental strains. These data demonstrate that during sCAR-Fc exposure, CVB3 can develop resistance against sCAR-Fc by single-amino-acid exchanges within the virus-receptor binding site, which, however, come at the expense of viral fitness.

**IMPORTANCE** The emergence of resistant viruses is one of the most frequent obstacles preventing successful therapy of viral infections, representing a significant threat to human health. We investigated the emergence of resistant viruses during treatment with sCAR-Fc, a well-studied, highly effective antiviral molecule against CVB infections. Our data show the molecular aspects of resistant CVB3 mutants that arise during repetitive sCAR-Fc usage. However, drug resistance comes at the price of lower viral fitness. These results extend our knowledge of the development of resistance by coxsackieviruses and indicate potential limitations of antiviral therapy using soluble receptor molecules.

## Abstract (precis)

The emergence of resistant viruses is one of the most frequent obstacles preventing successful therapy of viral infections, representing a significant threat to human health. We investigated the emergence of resistant viruses during treatment with sCAR-Fc, a well-studied, highly effective antiviral molecule against CVB infections. Our data show the molecular aspects of resistant CVB3 mutants that arise during repetitive sCAR-Fc usage. However, drug resistance comes at the price of lower viral fitness. These results extend our knowledge of the development of resistance by coxsackieviruses and indicate potential limitations of antiviral therapy using soluble receptor molecules.

## INTRODUCTION

Coxsackie B virus (CVB) infections are spread worldwide, and their clinical manifestations vary from asymptomatic infections or mild, cold-like symptoms to life-threatening conditions, such as pancreatitis, myocarditis, and encephalitis (1, 2). Newborns and children, in particular, risk a severe progression of the disease, and long-term sequelae may occur as well as chronic manifestations, like chronic myocarditis and type 1 diabetes (3, 4).

As a member of the picornavirus family, CVB are characterized by a positive-sense single-stranded RNA genome, located in a small nonenveloped viral capsid. The viral capsid possesses an icosahedral architecture and is composed of the four viral proteins (VP1 to VP4). VP4 is located at the inner capsid site associated with the viral genome (5). A small depression is located around each 5-fold axis, the so-called canyon, containing the virus receptor binding site (6). During infection, the virus interacts with the cellular coxsackievirus and adenovirus receptor (CAR) (7, 8). Cryo-electron microscopy (cryo-EM) studies have determined the amino acids within the capsid region responsible for the virus-receptor interaction, which allows the prediction of a footprint of CAR on the CVB surface (6, 9). After virus-receptor interaction at physiological temperature, CVB, like other picornaviruses, undergoes a specific structural reorganization, critical for cellular uptake and infection (6, 9, 10). Shortly after receptor interaction, the viral capsid expands and transitions to so-called altered (A) particles. These A-particles are noninfectious and lack both the VP4 protein and the ability to bind to the cognate virus receptor CAR. Formation of A-particles is also induced by the interaction of CVB with soluble forms of CAR (sCAR). These soluble receptors have a high binding affinity to CVB and efficiently neutralize bound virus (11, 12).

Soluble receptors have long been used to analyze virus-receptor interactions. When these interactions entail high inhibition efficiency and minimal side effects, their attractiveness as antiviral therapeutics increases. A virus-neutralizing effect of soluble receptor proteins has been described for several viruses, such as adenovirus, measles virus, human herpesvirus 6, cytomegalovirus (CMV), and human immunodeficiency virus (HIV) (13–16). Compared to these viruses, where the antiviral effect is attributed to reversible, competitive inhibition, the picornavirus-specific formation of A-particles leads to the irreversible neutralization of these viruses and, therefore, is much more efficient. Over the last decade, we and others have confirmed the antiviral activity of the soluble receptor protein, sCAR-Fc, where the extracellular part of CAR is fused to the constant domain of human IgG. The sCAR-Fc neutralizes all CVB serotypes (CVB1 to CVB6), and a prophylactic, as well as early therapeutic treatment, significantly inhibits CVB3-induced pancreatitis and myocarditis in mice (17–21). Recently, we have shown that not only various laboratory CVB strains but also 23 clinical strains are highly susceptible to sCAR-Fc-induced neutralization, isolated from patients with neurological or respiratory diseases (22).

Due to the very error-prone RNA-dependent RNA polymerase (RdRp), RNA viruses, such as picornaviruses, have a high mutation rate, resulting in genetically diverse viral populations, so-called quasispecies, and the rapid emergence of resistant viruses (23, 24). As shown for the broad-spectrum capsid binder, Pleconaril, resistant mutants can already exist within a population before treatment begins or rapidly emerge during treatment (25, 26). On the other hand, it has been proposed that viral mutants resistant to the antiviral effects of their soluble receptors would not emerge, because mutations that abrogate binding to the cognate virus receptor would be lethal (27). However, during treatment with soluble virus receptor traps, later investigations revealed the emergence of resistant viruses for HIV, CMV, and another member of the picornavirus family, poliovirus (PV) (28–31).

In this study, we investigated development of resistance of CVB3 against its highly effective soluble receptor molecule, sCAR-Fc. We show that during serial passage in the presence of sCAR-Fc, resistant CVB3 mutants emerge that are characterized by single-amino-acid exchanges in the virus-receptor recognition site. We also investigated the replication properties, stability, and receptor dependency of these mutants and demonstrated that sCAR-Fc resistance decreases viral fitness.

## RESULTS

### Resistant CVB3 Nancy mutants arise during repeated exposure to sCAR-Fc.

To assess whether resistant CVB3 mutants emerge during repeated exposure to sCAR-Fc, the laboratory strain CVB3 Nancy was serially passaged in HeLa cells after preincubation with 1 μg/ml sCAR-Fc. This amount represents an sCAR-Fc concentration that inhibited viral replication between 90% and 99% (22), allowing survival of enough virus progeny to enable passaging at a constant MOI. In two independent experiments, HeLa cells were subsequently infected after sCAR-Fc incubation, and replication was quantified after 24 h. The replication inhibition efficiency of sCAR-Fc was calculated relative to CVB3 Nancy replication after preincubation in the absence of sCAR-Fc. For serial passaging, viral progeny from each round of sCAR-Fc treatment was incubated again, with or without sCAR-Fc, and the efficiency of inhibition was calculated as previously explained.

**FIG 1:**
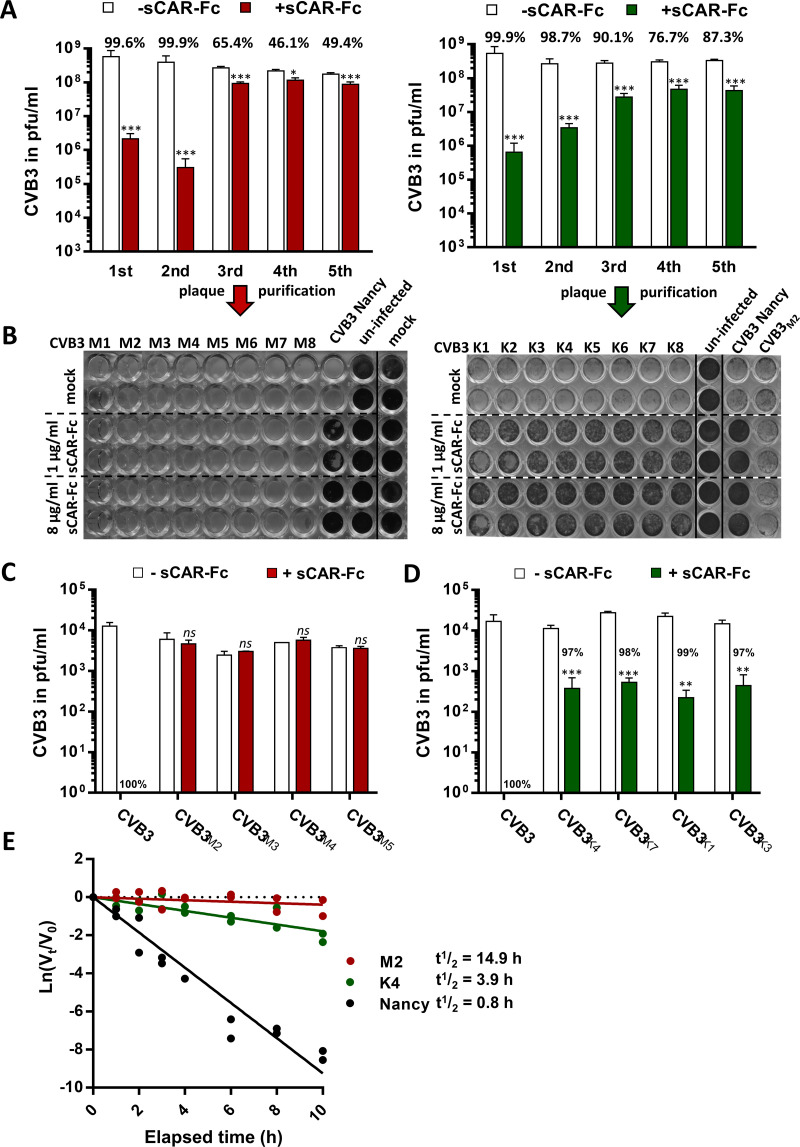
Resistant CVB3 Nancy mutants emerge after repetitive sCAR-Fc exposure. (A) Inhibition efficacy of sCAR-Fc during serial passaging of CVB3 Nancy with sCAR-Fc in two independent experiments. Virus was preincubated with 1 μg/ml sCAR-Fc at 4°C for 30 min, and HeLa cells were subsequently infected for 24 h. The amount of virus was determined by plaque assay, and inhibition efficacy was calculated as a percentage relative to virus replication without sCAR-Fc preincubation. Student’s *t* tests were performed. *, *P < *0.05; ***, *P < *0.001. (B) Eight clones were selected from the third round of sCAR-Fc passaging (A) by plaque purification, and antiviral efficacy of sCAR-Fc was analyzed in a cell-killing assay. HeLa cells were infected with each isolated clone (MOI, 0.1) after preincubation with 1 μg/ml, 8 μg/ml, or no sCAR-Fc (30 min, 4°C), and cells were stained 36 h p.i. with crystal violet. Cells infected with the wild-type CVB3 Nancy, uninfected cells, and mock-treated cells served as controls. (C) Isolated virus mutants from the first serial passaging experiment under sCAR-Fc treatment (A, left graph) are completely resistant to sCAR-Fc-induced neutralization. The plaque-purified CVB3 mutants CVB3_M2_ to CVB3_M5_ and 10,000 PFU of the original CVB3 Nancy strain were preincubated with 8 μg/ml sCAR-Fc for 30 min at 37°C, infected for 30 min at 37°C, and subsequently overlaid with agar. Plaques were counted 2 to 3 days after infection. Values are given as means ± SD from three independent experiments. Student’s *t* tests were performed. *ns*, not significant. (D) Isolated virus mutants from the second serial passaging experiment under sCAR-Fc treatment (A, right graph) are less susceptible to sCAR-Fc-induced neutralization. Plaque-purified CVB3_K_ clones were incubated with 8 μg/ml sCAR-Fc for 30 min at 37°C, and infectious virus was measured as described for panel C. The original CVB3 Nancy strain served as a control. Values are given as means ± SD from three independent experiments. Student’s *t* tests were performed. **, *P < *0.01; ***, *P < *0.001. (E) The loss of infectious CVB3 over time at 37°C in the presence of sCAR-Fc has first-order decay kinetics. Wild-type strain CVB3 Nancy and the sCAR-Fc resistant mutants CVB3_M2_ and CVB3_K4_ were incubated at 37°C with 32 ng/ml (0.25 nM) sCAR-Fc, and the conversion to noninfectious A-particles was analyzed over time by plaque assay. *V*_0_ is the concentration of infectious virus at the start of the incubation at 37°C, and *V_t_* is the amount of infectious virus that remains after the indicated time points. Virus infectious half-life was determined with the equation *t*_1/2_ = ln 0.5/−*k*, with *k* as the first-order rate constant.

In the first round, sCAR-Fc inhibited the replication of CVB3 Nancy by more than 99% (Fig. 1A). After the third round of sCAR-Fc incubation, the inhibitory effect significantly declined in both experiments, but each in a different manner. In the first experiment, the inhibition of CVB3 replication suddenly fell from 99.9% to 65% (Fig. 1A, left graph), while in the second experiment the antiviral effect of sCAR-Fc declined in a stepwise manner from 99.9% in the first round to 76.7% after four rounds of sCAR-Fc incubation (Fig. 1A, right graph). During repeated exposure to sCAR-Fc, virus mutants arose that were less susceptible or even resistant to sCAR-Fc-induced neutralization.

Interestingly, for another commonly used CVB3 strain, CVB3-H3, no decrease in the antiviral efficacy of sCAR-Fc during serial passaging under various experimental conditions was observed (Table 1).

**TABLE 1:** No sCAR-Fc-resistant mutants emerge during serial passaging of CVB3-H3^*a*^

Round of sCAR-Fc treatment	Inhibition efficiency (%)			
	8 h replication		24 h replication	
	4°C	37°C	4°C	37°C
1	97 ± 1.5	97 ± 0.1	97 ± 0.7	97 ± 0.8
2	97 ± 2.6	99 ± 0.6	89 ± 0.8	98 ± 0.01
3	89 ± 9.6	99 ± 0.9	96 ± 1	98 ± 0.5
4	93 ± 2.3	99 ± 0.1	84 ± 6.5	96 ± 0.8
5	95 ± 0.5	98 ± 0.3	90 ± 1.9	97 ± 0.4

a Inhibition efficacy of sCAR-Fc during serial passaging of CVB3-H3. For replication for 8 h, HeLa cells were infected with 30,000 PFU (MOI, 0.1) of CVB3-H3 that had been preincubated with 0.5 μg/ml sCAR-Fc when incubated at 4°C or with 0.25 μg/ml sCAR-Fc when incubated at 37°C. Virus replication was assessed 8 h later by plaque assay. For replication for 24 h, HeLa cells were infected with 30,000 PFU (MOI, 0.1) of CVB3-H3, which had been preincubated with 1.0 μg/ml sCAR-Fc when incubated at 4°C or 0.5 μg/ml sCAR-Fc when incubated at 37°C. Virus replication was assessed 24 h later by plaque assay. Viral progeny resulting from the first round of sCAR-Fc treatment was used for the second round, again incubated with sCAR-Fc and propagated as described above. Inhibition efficacy was calculated in percentage relative to virus replication without sCAR-Fc preincubation. Values are given as means ± SD from two independent experiments.

For subsequent analysis, eight clones from each serial passaging experiment were isolated by plaque purification from cell lysates after the third round of sCAR-Fc incubation. At this time point of the experiment, the detection of reduced inhibition efficiency by sCAR-Fc indicated the emergence of resistant mutants (Fig. 1A). Cell-killing assays were performed to determine the susceptibility of the isolated clones, CVB3_M1_ to CVB3_M8_ from the first experiment and CVB3_K1_ to CVB3_K8_ from the second experiment, to sCAR-Fc. HeLa cells were infected with each clone after preincubation with 1 μg/ml or 8 μg/ml or without sCAR-Fc, and cell viability was assessed by crystal violet staining. While the cell-killing activity of the CVB3 Nancy wild type was significantly inhibited by 1 μg/ml sCAR-Fc and completely prevented by 8 μg/ml (Fig. 1B), none of the CVB3_M1_ to CVB3_M8_ isolated clones were inhibited by even the high-dose sCAR-Fc, as indicated by complete virus-induced cell lysis (Fig. 1B, left). On the other hand, the cell-killing assay of CVB3_K1_ to CVB3_K8_ showed a few living cells after preincubation with the lower-dose sCAR-Fc (1 μg/ml), while treatment with high-dose sCAR-Fc almost completely protected HeLa cells from virus-induced cell lysis (Fig. 1B, right). This indicates that in both serial passaging experiments involving sCAR-Fc treatment, CVB3 Nancy mutants arise with reduced susceptibility to sCAR-Fc. While CVB3_K_ clones seemed to be less susceptible to sCAR-Fc, the CVB3_M_ clones from the first experiment were completely resistant.

Next, we performed neutralization assays to quantify the remaining inhibition efficiency of sCAR-Fc more precisely. To this end, 10,000 PFU of each analyzed CVB3_M_ clone and CVB3_K_ clone was preincubated with a high concentration of sCAR-Fc (8 μg/ml) at 37°C. Interaction of sCAR-Fc with the virus at this physiological temperature immediately leads to the formation of noninfectious A-particles, thereby increasing the antiviral efficiency (11, 22). While the parental CVB3 Nancy strain was completely neutralized by sCAR-Fc (Fig. 1C and D), it did not impair the infectivity of the isolated clones CVB3_M2_ to CVB3_M5_, confirming their complete resistance. As observed in the cell-killing assay, the isolated CVB3_K_ clones showed a decreased susceptibility to sCAR-Fc-induced neutralization, but, in contrast to the CVB3_M_ clones, they were not completely resistant (Fig. 1D). The neutralization efficacy for all four analyzed CVB3_K_ clones was about 98%.

We next determined the long-term stability of sCAR-Fc-resistant mutants against sCAR-Fc-catalyzed conversion into A-particles. Therefore, we incubated one representative of each of the resistant mutants, CVB3_M2_ and CVB3_K4_, as well as the susceptible wild-type CVB3 Nancy, with sCAR-Fc over a period of about 10 h at 37°C. The sCAR-Fc-catalyzed loss of infectivity was quantified by determination of infectious particles using plaque assays. As shown in Fig. 1E, the parental CVB3 Nancy, as a highly sCAR-Fc-susceptible strain, rapidly lost its infectivity with a calculated half-life (*t*_1/2_) of 0.8 h. Compared to short-term incubation with sCAR-Fc (Fig. 1B and C), where the CVB3_M_ clones revealed complete resistance, long-term sCAR-Fc incubation of the parental and mutant strains showed that CVB3_M2_ is not completely resistant to sCAR-Fc *per se*. However, the sCAR-Fc-triggered conversion of CVB3 to noninfectious particles is greatly slowed in the CVB3_M2_ mutant, with a calculated half-life of 14.9 h. Moreover, the half-life of the CVB3_M2_ mutant was much longer than the half-life of CVB3_K4_ (*t*_1/2_ = 3.9 h), providing additional evidence for the higher resistance of the CVB3_M_ mutants compared to that of the CVB3_K_ mutants (Fig. 1E). In conclusion, during repeated incubation of CVB3 Nancy with low concentrations of sCAR-Fc, viral mutants emerge that are less susceptible or nearly completely resistant to sCAR-Fc. Our data also revealed that CVB3_M2_ is completely resistant to short-term sCAR-Fc incubation, while, during long-term incubation, a greatly slowed conversion of CVB3_M2_ to noninfectious particles occurs.

### A single mutation in the virus-receptor recognition site is sufficient for sCAR-Fc resistance.

**FIG 2:**
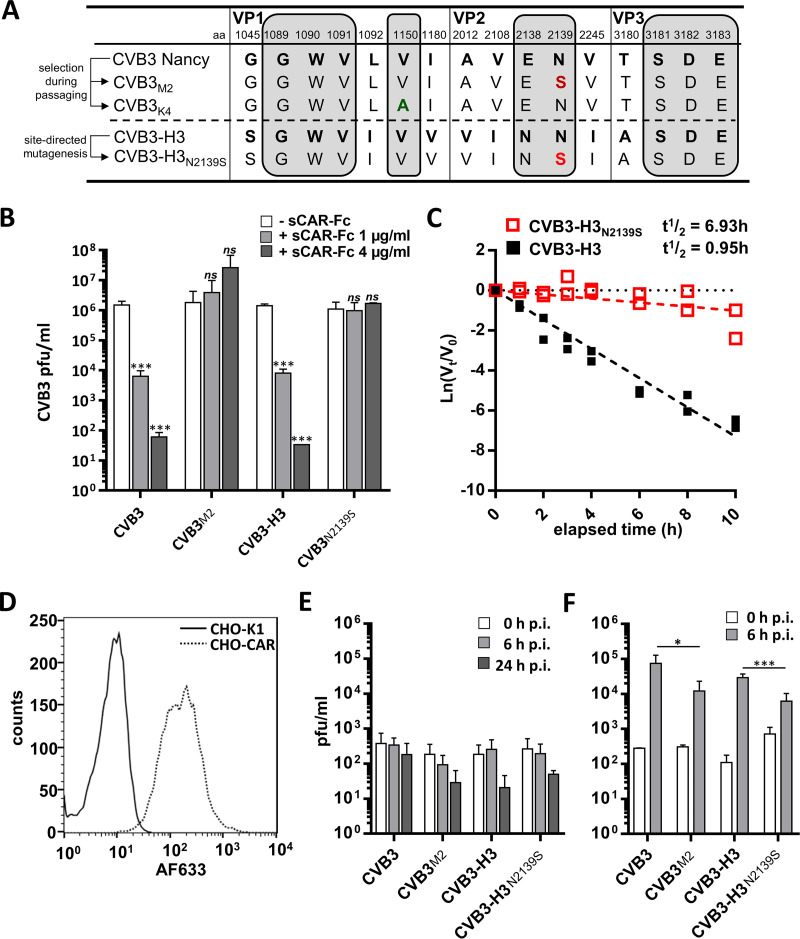
Single mutation within the CAR binding region of the virus capsid leads to sCAR-Fc resistance. (A) Sequence polymorphism of the capsid proteins from CVB3 strains H3 and Nancy (boldface). The first digit of the amino acid description indicates the capsid protein, while the following three numbers identify the amino acid position. The amino acid substitutions in the sCAR-Fc-resistant mutants CVB3_M2_ (N2139S, red) and CVB3_K4_ (V1150A, green) are shown. The amino acids that are thought to be involved in CAR binding are shaded gray. All analyzed clones within the same passaging experiment revealed the same amino acid exchange. (B) Impact of the amino acid substitution in VP2 (N2139S) on sCAR-Fc susceptibility. The mutation was inserted into the sCAR-Fc-sensitive CVB3-H3 cDNA clone by *in vitro* mutagenesis, and sCAR-Fc susceptibility of the resulted CVB3-H3_N2139S_ was compared to that of the parental strain CVB3-H3. Various amounts of sCAR-Fc were preincubated with 10^6^ PFU of each virus for 30 min at 37°C, and cells then were infected for 30 min at the same temperature and subsequently overlaid with agar. Plaques were counted 2 to 3 days postinfection. The sCAR-Fc-resistant clone CVB3_M2_, isolated during serial passaging under exposure to sCAR-Fc, and the parental CVB3 Nancy strain served as additional controls. Values are given as means ± SD from three independent experiments. Student’s *t* tests were performed. *ns*, not significant; ***, *P < *0.001. (C) Loss of infectious CVB3 by N2139S in the presence of sCAR-Fc represented as the first-order decay curve. CVB3-H3 and the sCAR-Fc-resistant mutant CVB3-H3_N2139S_ were incubated at 37°C with 32 ng/ml (0.25 nM) sCAR-Fc, and conversion to noninfectious A-particles was analyzed over time by plaque assay (in duplicates). *V*_0_ is the concentration of infectious virus at the start point of incubation at 37°C, and *V_t_* is the amount of infectious virus that remains after the indicated time points. Virus infectious half-life was determined with the equation *t*_1/2_ = ln 0.5/−*k*, with *k* as the first-order rate constant. Results are shown from two independent experiments. (D) CAR expression on CHO-K1 cells and CHO-CAR cells determined by flow cytometry. CAR on the cell surface was detected by anti-CAR antibody (RmcB) and AF633-labeled secondary antibody. (E and F) The infection of sCAR-Fc-susceptible strains and resistant variants depends on CAR. (E) CAR-negative CHO-K1 cells were inoculated with sCAR-Fc-resistant mutants (CVB3_M2_ and CVB3-H3_N2139S_) and the parental strains (CVB3 and CVB3-H3) (MOI, 1) and incubated for 0 h, 6 h, and 24 h. Virus titers were determined by plaque assay. Means ± SD are displayed from three independent experiments, each performed in duplicate. (F) CAR-expressing CHO-CAR cell were infected with each of the analyzed CVB3 strains (MOI, 2), and virus titers were determined 6 h p.i. by plaque assay. Means ± SD are displayed from two independent experiments, each performed in triplicates. Student’s *t* tests were performed. *, *P < *0.05; ***, *P < *0.001.

To gain insight into underlying mechanisms of emerging sCAR-Fc resistance, the complete capsid region of the isolated resistant clones was sequenced and compared to that of the CVB3 Nancy strain. As expected from the different phenotypes, the virus mutants from the first (CVB3_M_) and second (CVB3_K_) serial passaging experiments showed nucleotide changes that result in different single-site amino acid substitutions (Fig. 2A). All analyzed clones from one passaging experiment revealed the same substitution. The completely resistant CVB3_M_ clones are mutated in the coding sequence of the VP2 protein, leading to an amino acid exchange from Asn (N) to Ser (S) at position 139, here referred to as N2139S. The less susceptible CVB3_K_ clones are mutated at amino acid residue 150 in the VP1 protein, changing Val (V) to Ala (A), here referred to as V1150A. Both amino acids, N2139 and V1150, are predicted to be located within the receptor footprint of the viral capsid (6, 9), most likely directly affecting the virus-receptor interaction.

In further investigations, we focused on the completely resistant CVB3_M_ clones and examined whether the single-amino-acid exchange in VP2 is responsible for resistance to sCAR-Fc. To analyze whether substitution N2139S indeed induces complete sCAR-Fc resistance, we changed the coding sequence for amino acid N2139 by site-directed mutagenesis into the coding sequence for serine (S) in the cDNA clone of CVB3-H3, generating CVB3-H3_N2139S_. A replication inhibition assay demonstrated that both viruses with the N2139S substitution, CVB3_M2_ selected during sCAR-Fc exposure of CVB3 Nancy and CVB3-H3_N2139S_ generated by mutagenesis of CVB3-H3, were completely resistant to sCAR-Fc, whereas both parental viruses were efficiently inhibited (Fig. 2B). Moreover, as shown for CVB3_M2_, long-term incubation of CVB3-H3_N2139S_ with sCAR-Fc revealed a greatly slowed and less pronounced transition to noninfectious particles over time compared to the sCAR-Fc-susceptible parental strain CVB3-H3 (Fig. 2C). Altogether, these data demonstrate that the N2139S mutation, acquired by CVB3_M2_ during viral passaging in the presence of sCAR-Fc, indeed determines the development of resistance to sCAR-Fc.

Given the complete resistance of CVB3_M2_ and CVB3-H3_N2139S_ to sCAR-Fc-induced conversion to noninfectious A-particles (11, 12), we asked whether CVB3 strains with the N2139S mutation still use CAR as a cellular receptor for binding and uptake. To address this issue, we infected the CAR-deficient cell line CHO-K1 (Fig. 2D) with the resistant mutants CVB3_M2_ and CVB3-H3_N2139S_ as well as with their parental strains CVB3 Nancy and CVB3-H3. Determination of infectious viruses by plaque assay directly after incubation (0 h postinfection [p.i.]) and 24 h later (24 h p.i.) showed that the sCAR-Fc-resistant mutants, just as the parental strains, were unable to infect CHO-K1 cells lacking CAR (Fig. 2E). Additional infection experiments with CHO-K1 cells stably expressing CAR on the cell surface (Fig. 2D, CHO-CAR) revealed a markedly increased virus concentration 6 h after infection for all virus strains (Fig. 2F), illustrating that both the parental viruses and the sCAR-Fc-resistant variants use CAR as a cellular receptor, independent of the efficiency of their neutralization by sCAR-Fc.

### The sCAR-Fc resistant viruses exert reduced viral fitness.

**FIG 3:**
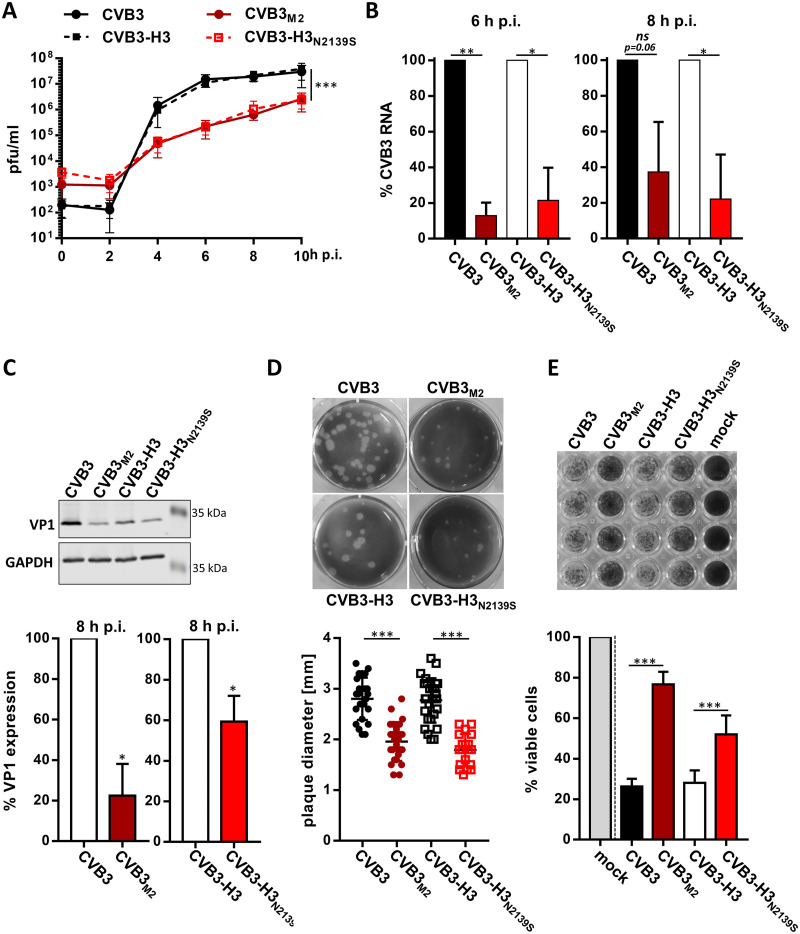
CAR-resistant CVB3 mutants show restricted replication compared to the susceptible wild types. (A) Shown is the analysis of virus replication of wild-type and sCAR-Fc-resistant mutants by one-step growth curve in HeLa cells. Cell monolayers were infected at an MOI of 2 (30 min, 37°C) with the indicated CVB3 isolates. After inoculation, virus was removed, monolayers were washed twice with PBS, and culture medium was added. At the indicated time points after infection, cells were frozen and virus was quantified by plaque assay. Each time point is the average of triplicates from two independent experiments (means ± SD). Two-way ANOVA was used for statistical analysis to compare the growth curves of the sCAR-Fc-resistant CVB3 mutants with the respective parental strains. ***, *P < *0.001. (B) Determination of viral RNA genome replication of wild-type and sCAR-Fc-resistant mutants was quantified by real-time RT-PCR. HeLa cells were infected as described for panel A, and CVB3-RNA was quantified by real-time RT-PCR. Expression data at 6 and 8 h after infection were normalized to the expression level of the housekeeping gene HPRT and the RNA level after infection (0 h p.i.) for each virus strain by following the 2^−ΔΔCT^ method. RNA levels of the sCAR-Fc-resistant mutants are shown as means ± SD relative to the respective wild-type strain. Results are shown from three independent experiments. One-sample *t* tests were performed. *ns*, not significant; *, *P < *0.05; **, *P < *0.01. (C) Quantification of viral protein expression by Western blotting. HeLa cells were infected at an MOI of 0.1 with the indicated CVB3 isolates, and the amount of the viral capsid protein VP1 was quantified 8 h later. GAPDH served as a loading control. The upper panel shows a representative Western blot of three independent experiments, quantified and summarized in the lower graph. One-sample *t* tests were performed. *, *P < *0.05. (D) Plaque sizes of the indicated CVB3 variants were determined by plaque assay on HeLa cells. The upper panel shows one representative image of virus plaques 2 days after infection. Plaque diameters of CVB3 (*n* = 24), CVB_M2_ (*n* = 25), CVB3-H3 (*n* = 27), and CVB3-H3_N2139S_ (*n* = 19) were measured in two independent experiments, and results are shown in the lower graph as means ± SD. Student’s *t* tests were performed. ***, *P < *0.001. (E) The virus-induced cytopathic effect was examined by cell-killing assay. HeLa cells were infected at an MOI of 0.1, and cells were stained 24 h p.i. with crystal violet. The upper panel shows one representative image of the cell-killing assay, and the lower graph represents the densitometric quantification of two independent experiments, each in quadruplicates. Results are shown as means ± SD in percentage relative to mock-infected cells (100%). Student’s *t* tests were performed. ***, *P < *0.001.

**FIG 4:**
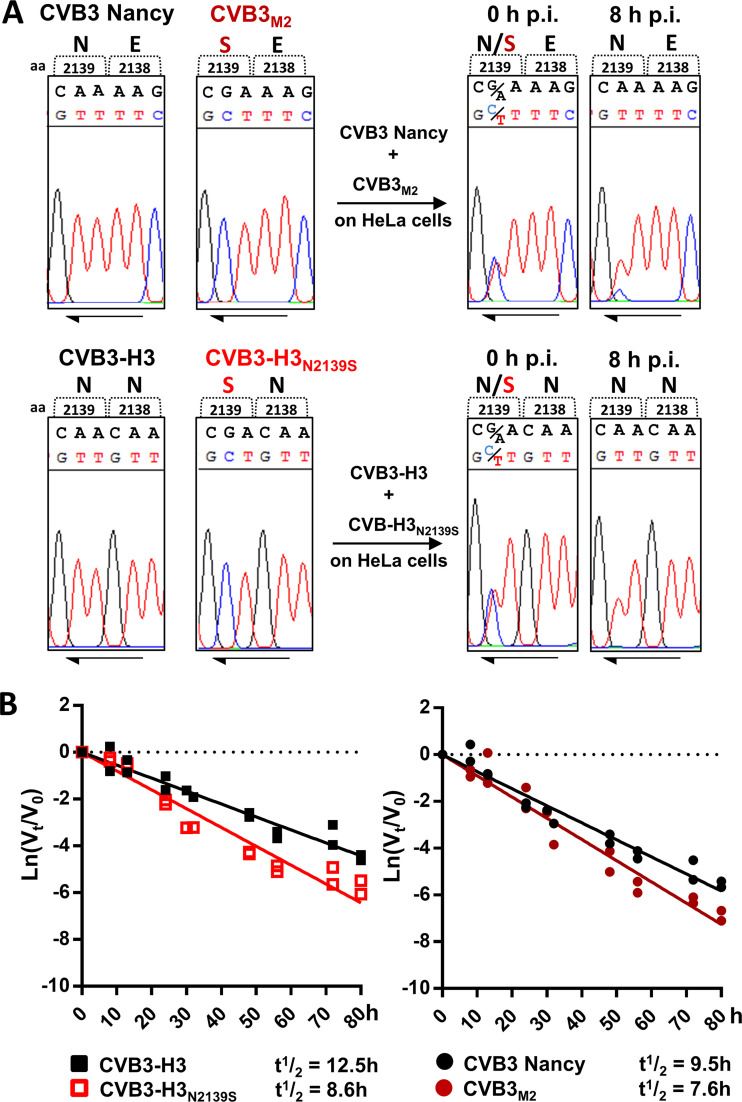
Wild-type strains surpass the sCAR-Fc-resistant CVB3 mutants in replication competition and virus stability. (A) Viral fitness was assayed in a replication competition assay. HeLa cells were infected with equal amounts of each virus, the sCAR-Fc-resistant mutants (CVB3_M2_ and CVB3-H3_N2139S_), and their respective susceptible paternal strains (CVB3 Nancy and CVB3-H3) (MOI, 1), and viral RNA was analyzed 0 h and 8 h postinfection. Sequence analysis revealed the ratio of each virus genome by detection of the nucleotide responsible for the amino acid substitution (N to S at position 2139) in CVB_M2_ and CVB3-H3_N2139S_. (B) Loss of CVB3 infectivity over time at 37°C. Wild-type strains CVB3-H3 and Nancy and the sCAR-Fc-resistant mutants were incubated at 37°C for the indicated periods, and conversion to noninfectious particles was analyzed. *V*_0_ is the concentration of infectious virus at the start point of incubation at 37°C, and *V_t_* is the amount of the remaining infectious virus, determined by plaque assay. Virus infectious half-life was determined with the equation *t*_1/2_ = ln 0.5/−*k*, with *k* as the first-order rate constant. Results are shown from two independent experiments.

Having shown that N2139S amino acid substitution does not prevent CAR-mediated virus uptake, the replication of both sCAR-Fc resistant mutants, CVB3_M2_ and CVB3-H3_N2139S_, was compared to those of their respective parental wild-type viruses in one-step growth curves in HeLa cells. Both resistant virus mutants replicated with slower kinetics and generated fewer progeny than their respective parental strains (Fig. 3A), confirming results obtained in CHO-CAR cells (Fig. 2F). Complementarily, RNA replication (Fig. 3B) and expression of viral proteins (Fig. 3C) were impaired for the resistant mutants. Both mutants had a diminished plaque size compared to their parental viruses when assayed on HeLa cells 2 days p.i. (Fig. 3D). As a consequence of the decreased viral replication, resistant CVB3 mutants were less cytopathic (Fig. 3E). Moreover, virus competition assay confirmed that the resistant mutants are inferior to their parental strains in terms of their replication efficiency (Fig. 4A). In fact, the sequencing chromatograms 8 h after infection with initial equal amounts of CVB3/CVB3_M2_ or CVB3-H3/CVB3-H3_N2139S_ (0 h p.i.) show that the wild-type virus sequences are overwhelmingly prevalent, while the mutated nucleotide, inducing the amino acid substitution Asn/N to Ser/S, is minimally present (CVB3/CVB3_M2_) or completely absent (CVB3-H3/CVB3-H3_N2139S_). These results point out that the amino acid substitution N2139S in CVB3_M2_ and CVB3-H3_N2139S_ generated a virus variant with an inferior ability to replicate.

Besides replication efficiency, the infectious half-life of a virus at physiological temperature is another indicator for viral fitness (32). At 37°C, dependent on the stability of individual strains, CVB3 shows a spontaneous transition into noninfectious particles, which is independent of its binding to the cognate receptor. Analysis of virus decay for the parental strains at 37°C revealed slight differences in virus stability between CVB3 Nancy and H3. More importantly, a comparison of sCAR-Fc-resistant mutants, CVB3_M2_ and CVB3-H3_N2139S_, with their parental viruses demonstrated a decreased half-life for sCAR-Fc-resistant strains, indicating faster decay of these mutant viruses (Fig. 4B) over an 80-h observation period. CVB3-H3, with a half-life of 12.5 h, was the most stable strain, but the amino acid substitution N2139S (CVB3-H3_N2139S_) decreased the stability to a half-life of 8.6 h. CVB3 Nancy had an infectious half-life of 9.5 h that dropped further after evolving into the sCAR-Fc-resistant mutant CVB3_M2_ (*t*_1/2_ = 7.6 h). These data indicate that amino acid substitution N2139S leads to reduced viral fitness by decreasing both virus stability and replication.

## DISCUSSION

Initially, it was suggested that viruses resistant to soluble receptors could not emerge, because mutations that interfere with virus-receptor binding would compromise the ability of the virus to infect host cells, leading to its rapid extinction from the viral population (27). In this study, we investigated the development of resistance of CVB3 against the soluble receptor molecule sCAR-Fc, which has been suggested as a potent antiviral compound against various CVB serotypes. Contrary to the assumption that soluble receptor-resistant viruses cannot evolve, we isolated different sCAR-Fc-resistant CVB3 mutants after *in vitro* serial passaging under low-dose sCAR-Fc exposure. It is particularly remarkable that resistant mutants could emerge among picornaviruses, because, on top of the simple competition for receptor binding sites, the sCAR-Fc-induced formation of noninfectious A-particles potentiates the antiviral effect of soluble virus receptors (11, 22). On the other hand, the low fidelity of RNA virus polymerases leads to very error-prone replication, contributing to the generation of high population diversity, which facilitates adaption of the virus to its current environment (33). Here, we report on two different mutations in the capsid proteins of CVB3, which were responsible for decreased susceptibility (V1150A, CVB3_K_ clones) or nearly complete resistance (N2139S, CVB3_M_ clones) of CVB3 to sCAR-Fc. Cryo-EM reconstructions predict that the identified amino acid exchanges that mediate their resistance are located within the CVB3-CAR interaction footprint. Hence, the defined mutations in the viral capsid proteins are most likely involved in the interaction of the virus with its cellular receptor CAR (6, 9), explaining their major impact on sCAR-Fc susceptibility. In accordance with our findings, the mutations in the majority of PV mutants known to be resistant to soluble PV receptor (PVR), and also Pleconaril-resistant CVB, were detected in the virus-receptor interaction site, verifying the biological relevance of this area for resistance development against binding inhibitors (25, 26, 30).

Interestingly, the position mutated in CVB3_M2_, N2139, has been found to be one of the amino acids contributing to the CVB3-CAR footprint. It is the only conserved amino acid in the otherwise highly variable VP2 puff region among different CVB serotypes (9). The conserved nature of N2139 was also shown by next-generation sequencing of CVB3 during long-term passaging in cell culture studies. During 40 passages, no minor or major variants with amino acid exchange in position 2139 were identified (33). Given the nearly complete resistance of the viruses CVB3_M2_ and CVB3-H3_N2139S_ investigated here_,_ N2139 seems to be especially important for receptor binding and uptake by A-particle formation (9).

During this study, resistant mutants were isolated during serial passaging of the CVB3 Nancy wild type but not with CVB3-H3. Sequence comparison of the two strains revealed eight amino acid differences in the capsid region. Although not all of these sequence differences are necessary to allow manifestation of complete resistance, as proven by insertion of N2139S into CVB3-H3, it is conceivable that the Nancy genotype somehow supports the development of sCAR-Fc resistance, whereas CVB3-H3 does not. The sequence differences in position VP3 residue 180 of CVB3-H3 (A3180) and CVB3 Nancy (V3180) have previously been described as influencing the stability of the virus and probably affect receptor binding and capsid expansion (32). Furthermore, the leucine at position 1092, detected in CVB3 Nancy but not in CVB3-H3, is associated with lower viral stability. This polymorphism is, in general, rarely found among clinical CVB isolates but accumulates in the laboratory strains and strains with resistance to antiviral drugs. This indicates that a less stable virus, such as CVB3 Nancy, is favored under the selection conditions present *in vitro* (32, 34). The VP2 residue 138, located right next to the amino acid that can confer resistance to sCAR-Fc (N2139S), seems to be of further interest. CVB3 Nancy with glutamic acid (E) in this position efficiently binds to the coreceptor DAF, while CVB3-H3, with asparagine (N) in this position, does not measurably bind to DAF (35, 36). Thus, the interaction with coreceptors, such as DAF, appears to support the evolution and maintenance of resistant viruses by partially compensating for a decreased binding affinity to CAR.

CAR and sCAR are believed to be bound by CVB3 in the same way, so it is astonishing that the sCAR-Fc resistant mutants CVB3_M2_ and CVB3_N2139S_ are still able to infect cells using CAR. Nevertheless, comparing the replication of the sCAR-Fc-susceptible CVB3 strains and the resistant viruses bearing the N2139S substitution, we observed decreased viral protein expression, RNA multiplication, and production of viral progeny for the sCAR-Fc-resistant strains, leading to smaller plaque size and a diminished virus-induced cytotoxicity. A virus competition assay revealed superior replication properties of the wild-type strains CVB3 Nancy and CVB3-H3 compared to their respective N2139S mutants. This finding differs from a previous observation in PV mutants that develop resistance against their soluble receptor. Here, virus propagation was not affected despite decreased virus receptor binding affinity (30). Both soluble receptor-resistant picornavirus mutants, PV and CVB3, still use their original receptor for cell infection (30). We assume that sCAR-Fc resistance is mainly based on delayed or inefficient transition to A-particles, an obligate stage during viral uptake into the host cell. This is supported by our virus/sCAR-Fc decay curves, where the mutants show a slowed transition to noninfectious particles. In addition, the one-step growth curves revealed up to 10-fold more cell-bound infectious CVB3_M2_ and CVB3-H3_N2139S_ directly after virus incubation, while the cellular binding of the virus was similar for the mutant and parental strains (data not shown). This indicates that the parental viruses efficiently converted to A-particles during cellular uptake, while, as reflected by the larger amount of infectious viruses at this stage, the transition process was inefficient for the mutant viruses. Our results indicate an inefficient conversion of the mutant strains to noninfectious A-particles by both soluble and membrane-bound CAR. Thus, it seems conceivable that the restricted replication of the mutant strains CVB3_M2_ and CVB3-H3_N2139S_, as indicated by diminished RNA multiplication, viral protein expression, and less viral progenies, is mainly a result of the slowed infection of the host cells.

Additional virus binding to coreceptors like DAF or heparan sulfate (37) or other unidentified receptors (18) could explain the existence of virus mutants with impaired CAR-related uptake capacities. It has been shown that DAF interaction increases the CVB3 infection in CaCo cells, where CAR is located in the tight junctions and, therefore, is hard for CVB3 to access (38). Therefore, binding to DAF or other coreceptors might compensate for the compromised CAR-induced uptake mechanism by continuously facilitating the accessibility of the virus to CAR.

In our analysis, we observed that the sCAR-Fc-resistant CVB3 mutants are slightly less stable with respect to their conversion to noninfectious particles than their respective parental strains. While greater stability is generally a useful trait for CVB3, it has been shown that this is not always the case (32). Under optimal cell culture conditions, the survival of weaker virus mutants or viruses with impaired fitness may be facilitated by the high abundance of virus receptors or additional molecules for virus binding (32). However, with slowed cellular uptake, restricted replication, and decreased stability *in vitro*, the virulence and pathogenicity of the sCAR-Fc-resistant mutants *in vivo* are questionable. Previous studies have shown that sCAR-Fc can be successfully employed for treatment of CVB3 infections *in vivo*, with no sign of the development of resistance (17, 20, 21). While we found sCAR-Fc-resistant isolates can emerge under our laboratory conditions, it remains unclear whether such sCAR-Fc-resistant isolates could emerge under clinical conditions. Should that prove to be the case, it would be important to better understand the limitations of sCAR-Fc as an anti-CVB therapeutic.

## MATERIALS AND METHODS

### Cells and viruses.

HeLa cells were cultured in minimum essential medium supplemented with 5% fetal calf serum (FCS), 1% penicillin-streptomycin (Merck KGaA, Darmstadt, Germany) 1% nonessential amino acids (NEAA; ThermoFisher Scientific, Waltham, MA, USA), and 2% HEPES buffer (ThermoFisher Scientific). HEK293T cells were cultured in high-glucose Dulbecco’s modified Eagle’s medium (DMEM; ThermoFisher Scientific) supplemented with 10% fetal calf serum and 1% penicillin-streptomycin (Merck KGaA). The CAR- and DAF-negative Chinese hamster ovary cell line (CHO-K1) was cultured in Ham’s F-12 medium (Lonza, Basel, Switzerland) supplemented with 10% FCS, 1% penicillin-streptomycin.

CHO-CAR cells stably expressing human CAR (hCAR) were generated by lentiviral vector transduction followed by puromycin selection. The hCAR sequence was inserted in the EcoRI/NheI-digested vector plasmid pLMJ1-EGFP (a gift from David Sabatini; Addgene plasmid number 19319, http://n2t.net/addgene:19319; RRID Addgene_19319 [39]). After validation of the inserted sequence, pLMJ1-hCAR was transfected into human embryonic kidney cells (HEK) 293T cells together with the lentiviral packaging plasmids psPAX2 and pMD2.G using PEI (polyethylenimine) Max (Polysciences, Warrington, PA, USA) as the transfection reagent. Both plasmids were a gift from Didier Trono (Addgene plasmid number 12260, http://n2t.net/addgene:12260, RRID Addgene 12260, and plasmid number 12259, http://n2t.net/addgene:12259, RRID Addgene 12259, respectively). Cell culture supernatant containing lentiviral vectors was collected 72 h after transfection, sterile filtered, and used for transduction of CHO-K1 cells. Medium containing lentiviral vectors was exchanged 6 h after transduction with fresh medium. Selection of successfully transduced cells was started 4 days posttransfection (p.t.) with 15 μg/ml puromycin (Merck KGaA), and single-cell clones were isolated after a period of 10 days under puromycin treatment.

CAR expression was verified by fluorescence-activated cell sorting analysis. Cells were stained using rabbit-anti-CAR antibody H-300 (Santa Cruz Biotechnology, Santa Cruz, CA, USA) and anti-rabbit-AF633 antibody (ThermoFisher Scientific). After the final wash step, the cells were analyzed with a FACSCalibur (BD Biosciences, Franklin Lakes, NJ, USA), and results were evaluated using the software FlowJo version 10.

The analysis of emerging sCAR-Fc-resistant mutants was carried out with the CVB3 laboratory strains Nancy and H3. CVB3 Nancy (VR-30) was obtained from the ATCC and propagated in HeLa cells. CVB3-H3 was generated by transfection of HEK293T cells with the cDNA-containing plasmid pBKCMV-H3 (kindly provided by Andreas Henke, Institute of Virology and Antiviral Therapy, Jena University Hospital, Friedrich Schiller University, Jena, Germany). HEK293T cells grown to 80% confluence were transfected using PEI Max. Between 18 and 24 h p.t., when cells showed distinct cytopathic effect, cell culture supernatants were harvested and the amount of infectious viral particles was measured by plaque assay after three freeze/thaw cycles, as previously described (18). For additional virus amplification, one T175 flask of HeLa cells was infected at a multiplicity of infection (MOI) of 5. Eight to 10 h after infection, when cells showed distinct cytopathic effects, the flask was freeze/thawed three times, the virus suspension was centrifuged at 5,000 × *g* for 20 min, and supernatant was aliquoted and stored at –80°C. The amount of infectious virus was quantified by plaque assay.

A cDNA plasmid clone of CVB3_N2139S_ was generated by *in vitro* mutagenesis using the CVB3-H3 plasmid pBKCMV-H3 and the primer CVB3-N2139S-sense 5′-GGG TGC TGT TTA GCG TTG C-3′ and CVB3-N2139S-rev 5′-GCA ACG CTA AAC AGC ACC C-3′. PCR was run using the Q5 high-fidelity DNA polymerase (New England Biolabs, Frankfurt am Main, Germany). Infectious virus was generated by transfection and amplification as described above. Virus genomic RNA was isolated from all virus stocks using TRIzol (ThermoFisher Scientific), and mutated amino acids were verified by sequencing.

### Production of sCAR-Fc.

To produce sCAR-Fc, HEK293T cells at a density of 90% were transfected with the sCAR-Fc-expressing plasmid pscAAVsCAR-Fc (15) using PEI Max (Polysciences). The medium was replaced with fresh medium containing 1% FCS 4 h p.t., and supernatant containing sCAR-Fc was harvested 72 h p.t. The concentration of sCAR-Fc was measured by enzyme-linked immunosorbent assay detecting human IgG-Fc (Bethyl Laboratories, Montgomery, TX, USA). The sCAR-Fc-containing cell culture supernatants were stored at –20°C until use.

### Quantification of CVB3 RNA by real-time RT-PCR.

For quantification of CVB3 RNA, total RNA was isolated with TRIzol, DNase I digested (ThermoFisher Scientific), and reverse transcribed by MLV-RT (Promega, Mannheim, Germany) in combination with random hexamer primers (Roche, Penzberg, Germany). Quantitative PCR was carried out using the TaqMan universal master mix (ThermoFisher Scientific) and the following primers and probes: human HPRT, 5′-AGT CTG GCT TAT ATC CAA CAC TTC G-3′ (forward), 5′-GAC TTT GCT TTC GGT CAG G-3′ (reverse), and 5′-TTT CAC CAG CAA GCT TGC GAC CTT GA-3′ (probe); CVB3, 5′-CCC TGA ATG CGG CTA ATC C-3′ (forward), 5′-ATT GTC ACC ATA AGC AGC CA-3′ (reverse), and 5′-FAM-TGC AGC GGA ACC G-MGB3′ (probe). A StepOnePlus real-time PCR system was used for quantitative PCR. Expression data were normalized to the expression level of the housekeeping gene HPRT and the RNA level after infection (0 h p.i.) for each virus strain by following the 2^−ΔΔCT^ method.

### Western blot analysis.

For SDS-PAGE, cells were scraped from cell culture plates and centrifuged, and cell pellets were lysed using 8 M urea buffer containing 1% Triton X-100, 0.1% SDS, 8 mM EDTA, 20 mM HEPES, 2 mM EGTA, 50 mM sodium fluoride, 5 mM sodium pyrophosphate, 2 mM sodium orthovanadate, 20 mM NEM, and cOmplete protease inhibitor cocktail (Roche). For detection of CVB3 capsid protein VP1 and glyceraldehyde-3-phosphate dehydrogenase (GAPDH), membranes were incubated with anti-VP1 antibody (3A8; Mediagnost) and anti-GAPDH antibody (ab181602; Abcam, Cambridge, UK). After incubation with secondary IRD680CW- or IRDye800CW-labeled antibodies, fluorescence was detected by the Odyssey CLx infrared imaging system (LI-COR Biosciences, Lincoln, NE, USA). Image Studio (LI-COR Biosciences) was used for quantitative densitometric analysis of protein expression. CVB3 VP1 values were normalized to GAPDH as an internal loading control.

### CVB3 quantification by plaque assay.

The amount of infectious CVB3 was determined on a monolayer of HeLa cells by plaque assay. Cell culture samples or agar pieces, including isolated virus in phosphate-buffered saline (PBS), were subjected to three freeze/thaw cycles and centrifuged to remove cell debris or agar. The number of infectious virus particles in the samples was quantified using a plaque assay, as previously described (18). Instead of neutral red, the plaques were stained using 0.5% MTT-PBS [3-(4,5-dimethylthiazol-2-yl)-2,5-diphenyltetrazolium bromide; Merck KGaA].

### One-step growth curve.

HeLa cell monolayers were infected in triplicates at an MOI of 2 (30 min, 37°C) for each virus. After inoculation, the virus was removed, cells were washed twice with PBS, and culture medium was added. Cells were frozen directly after incubation (0 h p.i.) or 2, 4, 6, 8, and 10 h after infection, and infectious virus was quantified by plaque assay.

### Virus neutralization assay.

Various amounts of virus were preincubated with or without sCAR-Fc at various concentrations at either 4°C or 37°C. If not otherwise described, after a preincubation period of 30 min, HeLa cells were infected for 30 min at 37°C and subsequently overlaid with agar. Emerging plaques were counted after 2 to 3 days, and percent neutralization efficiency was calculated relative to virus preincubated without sCAR-Fc. For virus plaque purification, single plaques were isolated under sterile conditions using a truncated 1,000-μl pipette tip. Agar pieces, including virus and cell debris, were resuspended in PBS, and infectious virus was quantified by plaque assay after three freeze/thaw cycles, as already described.

### Virus stability assay.

Virus stabilities and receptor-mediated conversion to noninfectious A-particles were assessed in terms of the first-order rate constant for inactivation at 37°C, as described previously in detail (9). Briefly, 1 × 10^7^ PFU of each analyzed virus was incubated without or with sCAR-Fc (32 ng/ml = 0.25 nM) at 37°C. Aliquots were taken at different time points, and the amount of infectious virus was quantified by standard plaque assay, as described above. The conversion of CVB3 to noninfectious A-particles with or without receptor was described as ln(*V_t_*/*V*_0_) over time, where *V_t_* is the concentration of infectious virus particles at the time (*t*) of incubation and *V*_0_ is the infectious virus concentration at the beginning. The curves were fitted by linear regression and slopes were calculated using GraphPad Prism 7.00 (GraphPad Software, San Diego, CA, USA). The decay over time was further expressed by calculated half-life (*t*_1/2_ = ln 0.5/−*k*, with *k* as the first-order rate constant).

### Virus replication inhibition assay.

Virus was preincubated with or without sCAR-Fc for 30 min at 4°C or 37°C, and HeLa cells were infected as described before. After infectious virus was removed, cells were washed with PBS and cultured for an additional 8 h to 24 h with HeLa cell growth medium.

### Virus competition assay.

HeLa cells were incubated with the virus pairs CVB3 Nancy/CVB3_M2_ and CVB3-H3/CVB3-H3_N2139S_ at an MOI of 1 of each virus for 1 h at 37°C. After two wash steps with PBS, RNA was isolated directly after incubation (0 h p.i.) and 8 h later (8 h p.i.). RNA was reverse transcribed using MLV-RT in combination with random hexamer primers. Viral genomic fragments containing the resistance-inducing mutation were amplified by PCR with the primers CVB3-seq1 (5′–CCG ATG CTT TGT CGA ACT TAG G-3′) and CVB3-seq2 (5′-CCT CTG TAC CAA CTT GTT GGA CC-3′). Fragments were analyzed by Sanger sequencing.

### Statistical analysis.

Statistical analysis of the results was performed with GraphPad Prism 7.00 (GraphPad Software). Results are shown as means ± standard deviations (SD) for each group. Unpaired Student's *t* test was used for two-group comparison. If values were normalized to an internal control, one-sample *t* test was applied. For multiple-group comparison, two-way analysis of variance (ANOVA) was performed. Differences were considered significant at a *P *value of *<*0.05.
